# An ICU Outbreak Due to Two Populations of Carbapenem-Resistant *Klebsiella pneumoniae* Isolates Belonging to ST11 and ST39 Types, Harbouring Double Carbapenemase Genes

**DOI:** 10.3390/microorganisms13122781

**Published:** 2025-12-06

**Authors:** Olga Koutsopetra, Sophia Vourli, Georgios Stravopodis, Sophia Hatzianastasiou, Stavros Dimopoulos, Themistocles Chamogeorgakis, Despina Tassi-Papatheou, Spyros Pournaras, Joseph Papaparaskevas

**Affiliations:** 1 Department of Microbiology, Medical School, National and Kapodistrian University of Athens, 11527 Athens, Greece; olga.koutsopetra@yahoo.com; 2 Clinical Microbiology Laboratory, “Attikon” University Hospital, National and Kapodistrian University of Athens, 12461 Chaidari, Greece; spournaras@med.uoa.gr; 3 Institute of Biosciences and Applications, National Center for Scientific Research “Demokritos”, 15341 Agia Paraskevi, Greece; vourli@bio.demokritos.gr; 4 Infectious Disease Committee, “Onassis” Hospital, 17674 Kallithea, Greece; g.stravopodis@onasseio.gr (G.S.); ypsos@hotmail.com (S.H.); 5 Surgical and Transplantation Intensive Care Unit, “Onassis” Hospital, 17674 Kallithea, Greece; s.dimopoulos@onasseio.gr (S.D.); t.chamogeorgakis@onasseio.gr (T.C.); 6 Microbiology Laboratory, “Onassis” Hospital, 17674 Kallithea, Greece; d.tasi@onasseio.gr

**Keywords:** *Klebsiella pneumoniae*, carbapenemase, multi-drug resistance, whole-genome sequencing

## Abstract

Carbapenem-resistant *Klebsiella pneumoniae* isolates harbouring double carbapenemases, from patients in a surgical and transplantation ICU, were investigated to better understand the dispersion of the pathogen. Twenty-three carbapenem-resistant *K. pneumoniae* isolates harbouring at least two different carbapenemases (by immunochromatography screening), were consecutively collected during a seven-month period from patients in a surgical and transplantation ICU. Identification and susceptibility testing were performed using the MALDI-TOF Vitek MS and the Vitek2 system (BioMerieux), respectively. Whole genome sequencing (WGS) was performed in an Illumina NextSeq2000 platform and MLST and resistome analysis of assembled genomes were performed by ResFinder, through the Center for Genomic Epidemiology platform. All isolates were resistant to ertapenem, imipenem, meropenem, and most to meropenem–varbobactam. Seventeen isolates belonged to the ST11 type and were positive for the OXA-48/NDM combination (by immunochromatography and NGS). Four isolates belonged to the ST39 type and were positive for the KPC/NDM combination (by immunochromatography and NGS). Finally, two isolates belonged to the ST258 type. One of them was positive for the OXA-48/KPC/NDM combination (by immunochromatography), but only *bla*_KPC_ was detected by WGS, and the second was positive for the OXA-48/KPC combination (by immunochromatography) and confirmed by WGS. This is the first report of an outbreak in Greece due to two simultaneous carbapenem-resistant populations harbouring double carbapenemases: a larger one comprising ST11 isolates harbouring the combination *bla*_NDM-1_/*bla*_OXA-48_, coupled by a smaller one comprising ST39 isolates harbouring the combination *bla*_KPC-2_/*bla*_NDM-1_. The implications of this particular situation regarding public health as well as intra-nosocomial infection prevention and control should be further monitored and studied.

## 1. Introduction

The genus *Klebsiella* comprises species that are frequent causes of infections, both nosocomial and community-associated, including bloodstream, respiratory tract, urinary tract, and surgical wound infections [1]. Nosocomial *Klebsiella* infections are caused mainly by the species *Klebsiella pneumoniae*, the most clinically significant species of the genus; to a much lesser degree, *K. oxytoca* has also been isolated from nosocomial infections. It is estimated that *Klebsiella* spp. cause 8% of all nosocomial bacterial infections in the United States and in Europe [2].

Resistance to carbapenems has increased considerably due to the acquisition of carbapenemase genes located in plasmids and mobile genetic elements, and carbapenem-resistant *K. pneumoniae* (CRKP) isolates are now frequently isolated from patients in ICUs around the world [3]. The clinically most significant types of carbapenemases comprise NDM, IMP, VIM, OXA-48, and KPC; production of these genes is currently the main molecular mechanism responsible for carbapenem resistance, whilst outer membrane protein (Omp) deficiency contributes as a secondary mechanism [4]. KPC production is the most prevalent mechanism on a global basis [5] and is often associated with the predominance of certain sequence types (STs), such as ST11 and ST39 [1].

During the last decade, double mechanisms of carbapenemases have been identified among CRKP, posing a new threat for the successful antimicrobial therapy of patients. For example, KPC-2/NDM-1-producing *K. pneumoniae* isolates have been identified in various settings around the world [1,6,7]. These isolates belong to different STs, although up to now they have not been detected among types considered to be hypervirulent. Nevertheless, these hypervirulent *K. pneumoniae* lineages are increasingly acquiring carbapenemase genes, representing a concerning shift toward convergent hypervirulent–resistant phenotypes [8].

In Greece up to now, double carbapenemase genes have been identified in certain hospitals, mostly as single isolates or smaller outbreaks [1]; however, it should be noted that the main mechanisms of resistance in Greek hospitals are either the KPC or the NDM ones.

In that respect a potential outbreak due to isolates possessing double carbapenemases may create a new threat in the hospital environment and it should be further investigated. Taking all that into account, and after the first isolation of a CRKP strain with two genes in our hospital, we investigated all CRKP isolated from patients at the Cardiac Surgery and Heart and Lung Transplantation ICU that were harbouring potential double carbapenemases with regard to the mechanisms of resistance and their sequence types.

## 2. Materials and Methods

### 2.1. Specimen Collection, Bacteria Selection, Identification, and Susceptibility Testing

Clinical specimens were consecutively collected from patients hospitalized at the 25-bed Cardiac Surgery and Heart and Lung Transplantation Intensive Care Unit (ICU) of the “Onassis” Hospital, during a seven-month period (October 2022 to April 2023). Specimens comprised blood, bronchial secretions, sputum, purulent wound exudates, urine, nasal swabs, and catheter tips.

Cultures were performed on MacConkey, 5% horse blood, chocolate and sabouraud agar plates, and thioglycolate broth (Bioprepare Microbiology, 19101, Keratea, Greece). Additionally, blood cultures were performed on a BacTAlert 3D automated blood culture system (bioMerieux, Marcy L’Etoile, France).

Identification of the isolates was performed using the VITEK^®^ MS PRIME Mass Spectrometry Microbial Identification System (bioMérieux). Susceptibility testing and Minimum Inhibitory Concentration (MIC) determination was performed using the VITEK^®^ 2 COMPACT automated susceptibility testing (bioMérieux) at the time of isolation, according to the manufacturer’s instructions, except for meropenem-vaborbactam which was performed using gradient MIC Test Strips (Liofilchem, Roseto degli Abruzzi 64026, Italy). Breakpoints and guidelines used for the study analysis were those of the EUCAST v15.0 document (available from https://www.eucast.org/clinical_breakpoints, accessed on 25 October 2025).

The presumptive presence of carbapenemases was investigated from the bacterial culture using the qualitative lateral flow immunoassay NG-Test^®^ CARBA-5 (NG—Biotech, Z.A., Courbouton, France) which detects and differentiates the five most prevalent carbapenemase types (NDM, IMP, VIM, OXA-48, and KPC).

Isolates were selected for inclusion in the study based on the following criteria:

1. Species identification as *Klebsiella pneumoniae.*
2. Resistance to imipenem and/or meropenem and/or ertapenem.
3. Presence by the lateral flow immunoassay of at least two or more carbapenemases.
4. One isolate per patient, the first one from any clinical specimen. If, however, a second isolate from a patient already enrolled, presenting with a different carbapenemase combination was detected, then this isolate was also included in the study as a different one.

### 2.2. Whole Genome Sequencing

Genomic DNA from a 24 h culture of the isolates on 5% horse blood agar plates was extracted using the QIAamp^®^ DNA Mini Kit (Qiagen, Hilden, Germany) and stored in −20 °C for further analysis.

DNA quantification was performed using a Qubit 2.0 Fluorometer (Life Technologies, Singapore). Whole genome sequencing was performed using the Illumina NextSeq 2000 (Illumina, San Diego, CA, USA), with DNA fragment libraries prepared using the Nextera XT kit (Illumina), following the manufacturer’s protocol.

The short sequencing reads, in FastQ format, were assembled into FastA format using the Unicycler Assembly Pipeline (Galaxy Version 0.5.0) via the online platform www.galaxy.com.

### 2.3. MLST and Phylogenetic Analysis

Isolates were analyzed using MLST (Software version: 2.0.9) via the Center for Genomic Epidemiology (CGE) online platform (https://www.genomicepidemiology.org last accesed on 1 July 2025), comparing the sequences of the genes *gap*A, *inf*B, *mdh*, *pgi*, *pho*E, *rpo*B, and *ton*B to reference sequences from the MLST database, assigning allele numbers based on sequence differences. 

Phylogenetic classification was performed using the MEGA11 platform (Software Version 11). The genome of *K. pneumoniae* isolate MGH-78578 (GenBank: CP000647.1) was used as a reference.

### 2.4. Resistome Investigation

The resistance genes of each strain were identified using the ResFinder platform (CGE, version: 4.5.0) via the CGE online service by comparing the assembled genome to known genomes in the database to identify resistance genes. A 90% ID threshold was set, representing the minimum percentage of identical nucleotides between the best-matching resistance gene in the database and the genome sequence. Additionally, a 60% minimum length threshold was selected, representing the minimum nucleotide overlap needed to count a gene as reliable.

Resistance genes were analyzed in strains phenotypically displaying a combination of resistance mechanisms. The resistance genes examined were linked to antibiotic groups such as beta-lactams, aminoglycosides, tetracyclines, macrolides, quinolones, fosfomycin, amphenicols, quaternary ammonium compounds, and folate pathway antagonists.

### 2.5. Ethical Considerations

A limited set of patients’ data was used anonymously (no clinical data was used, only data related to the bacterial isolates). The work has been approved by the hospital ethical committee.

## 3. Results

A total of 23 CRKP isolates were identified by MALDI and collected during the study period from 20 patients. Specimens included sterile sites (blood/urine), bronchial secretions from patients with pneumonia symptoms, and surgical wound infections (eight, eight and four cases, respectively), as well as non-sterile and screening sites (sputum, nasal swab and i.v. catheter tip, in one, one and one cases, respectively).

Based on the lateral flow assay screening results, the isolates were grouped into two main groups, 17 isolates harbouring the NDM/OXA-48 combination and four isolates harbouring the NDM/KPC combination. Finally, the combinations KPC/OXA-48 and OXA-48/KPC/NDM were detected among one and one isolates, respectively. Detailed results are depicted in Table 1.

The 23 CRKP strains showed resistance to multiple antibiotics, according to the susceptibility testing performed, and the detailed results of Table 2. More specifically, the 17 isolates of the dominant group were resistant to ertapenem, imipenem, and meropenem, whilst 11/17 (64.7%) were mostly highly resistant to the combination of meropenem–vaborbactam. The four isolates of the second group were all fully resistant to ertapenem, imipenem, meropenem, and meropenem–vaborbactam.

MLST results are presented in Table 3, respectively. All isolates belonged to main clonal group CG10011. The isolates of the dominant group (17/23, 73.9%) belonged to type ST11, whilst the four isolates of the second group belonged to type ST39. Finally, the two remaining isolates belonged to ST258.

**Figure 1:**
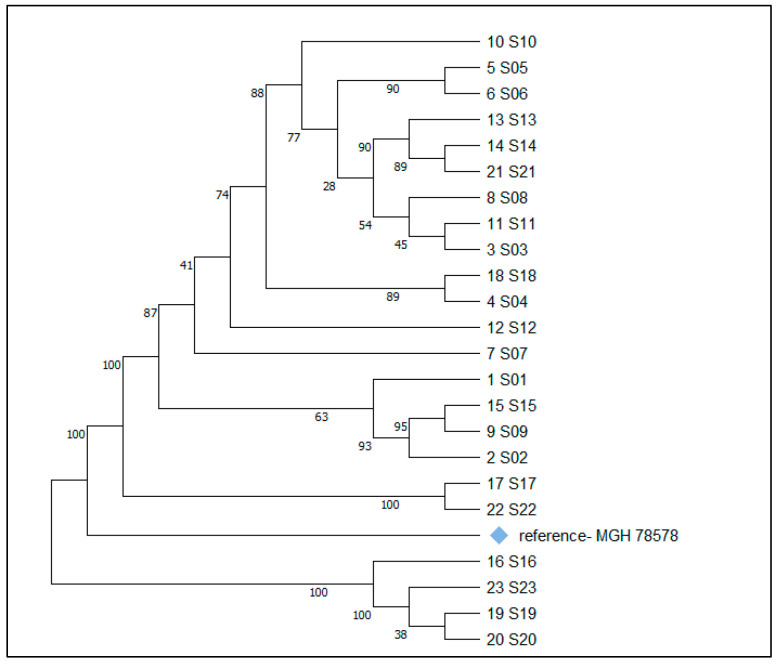
Phylogenetic tree of all 23 *K. pneumoniae* isolates.

The phylogenetic tree of the 23 isolates is shown in Figure 1.

Resistome analysis is depicted in Table 4. A total of 22 out of the 23 isolates were found to harbour two carbapenemase genes. More specifically, all 17 isolates assigned to ST11 were found to harbour the combination bla_NDM-1_/bla_OXA-48_, and all four isolates assigned to ST39 were found to harbour the combination bla_NDM-1_/bla_KPC-2_, whilst one of the two isolates assigned to type ST258 (isolate #22) was found to harbour the combination bla_KPC-3_/bla_OXA-48_, and the other one (isolate #17) was found to harbour only bla_KPC-3_. The carbapenem resistance genes results corresponded well with the lateral flow assays results, except for isolate #17.

In addition, resistome analysis indicated that the ST39 isolates harboured a considerably higher number of beta-lactamase genes, and more specifically the *bla*_SHV-79_, *bla*_SHV-89_, *bla*_SHV-40_, *bla*_SHV-85_, *bla*_SHV-56_, *bla*_CTX-M-15_, *bla*_TEM-1B_, *bla*_TEM-207_, *bla*_TEM-230_, *bla*_TEM-104_, *bla*_TEM-234_, *bla*_TEM-217_, *bla*_TEM-30_, and *bla*_TEM-198_ genes, whilst the ST11 isolates mostly harboured only the *bla*_OXA-1_, *bla*_SHV-182_, *bla*_CTX-M-15_, and *bla*_TEM-1B_ genes.

## 4. Discussion

The initial isolation of the first CRKP strain containing double carbapenemases (CRKP-DC) as shown by the lateral flow assay (isolate #8) indicated the onset of this particular outbreak and triggered the present study, which focused on strains identified as *K. pneumoniae* by MALDI, resistant to at least one of the antibiotics of the carbapenem group by MIC measurement, and found to possess at least two different carbapenemases by the initial test. Prior to strain #8 isolation, the sporadic CRKP that were detected in our ICU were mostly KPC(+) and were introduced through patient transfer from other hospitals.

The majority of the isolates included in the study (20/23) were from sterile sites, monomicrobial surgical site infections or bronchial specimens in patients with pneumonia, and only three isolates were from non-sterile sites or screening specimens.

Both the lateral flow assay and the sequencing data allocated the majority of the 23 CRKP-DC isolates into two main groups. The dominant one was Group A and consisted of isolates #1–15, 18, and 21, belonging to ST11, harbouring the combination *bla*_NDM-1_/*bla*_OXA-48_ and resistant to ertapenem, imipenem, meropenem, and the majority to meropenem–vaborbactam. In contrast, Group B consisted of isolates #16, 19, 20, and 23, belonging to ST39, harbouring the *bla*_KPC-2_/*bla*_NDM-1_ combination, all fully resistant to ertapenem, imipenem, meropenem, and meropenem–vaborbactam.

Although CRKP-DC have previously been described in Greece, CRKP-DC ST11 isolates are infrequent and only a single strain with similar characteristics has been reported previously in Thessaloniki, Greece [1]. In similar international studies from Italy [9] and Germany [10] strains identified with the combination *bla*_NDM-1_/*bla*_OXA-48_ belonged to types ST15 and ST383 in Italy and ST307 in Germany, but not ST11. Finally, in another study from Iran [11], CRKP-DC isolates expressing various combinations of *bla*_OXA-48_, *bla*_NDM-1_, and *bla*_NDM-7_ genes were reported, belonging not to a single but to various sequencing types, including ST11, ST893, ST147, ST392, and ST15, but not ST11.

In contrast to the infrequent isolation of CRKP-DC ST11 isolates, CRKP-DC ST39 isolates have previously been detected in our country in various settings [12,13,14] and the gene combinations that were detected were either the *bla*_KPC-2_/*bla*_VIM-1_ or the *bla*_KPC-2_/*bla*_NDM-1_. In other Greek hospitals, the CRKP-DC isolates harboured the *bla*_KPC-2_/*bla*_VIM-1_ genes and were clustered in various STs (ST39, ST147, ST323, ST258, ST3035, and ST340) [15] or the *bla*_NDM-1_/*bla*_KPC-3_ but belonged to the ST512 type and not the ST39 [16]. More than two genes were detected among isolates in a study conducted in Russia [3], in which four ST39 CRKP strains were found to express three carbapenemase genes simultaneously, *bla*_KPC-2_/*bla*_NDM-1_/*bla*_OXA-48_.

Two isolates (#17 and 22) in the present study belonged to type ST258 and harboured the single gene *bla*_KPC-3_ and the gene combination *bla*_KPC-3_/*bla*_OXA-48_, respectively. CRKP-DC strains of the ST258 type have been identified in Larissa, Greece [15], where simultaneous expressions of *bla*_KPC_ and *bla*_VIM_ genes were observed, although there were also ST258 strains in which only the *bla*_KPC_ gene was detected, as was the case in our study. The presence of two carbapenemases in ST258 *K. pneumoniae* was observed also in one isolate carried *bla*_KPC-2_ and *bla*_VIM-4_ in a New York City Hospital [17].

In that respect, to our knowledge, the situation in our hospital was unique compared to other Greek settings, as the clonal spread of the ST11 CRKP-DC isolates dominated the outbreak within the ICU and presented a novel epidemiological situation that needed continuous monitoring. The clonal spread of the ST11 CRKP-DC isolates begun in October 2022 and continued up to January 2023, and then it was replaced by the clonal spread of the ST39 CRKP-DC isolates up to April 2023, when this outbreak was finally eliminated as a result of the corrective measures that were implemented.

The majority of the isolates were resistant and/or highly resistant to the antimicrobial meropenem-vaborbactam. Among ST11 isolates there were a few susceptible ones, although no differences regarding the resistome analysis were detected between the susceptible and the resistant ST11 isolates. Of interest, however, is that the isolates that were considered susceptible to this antimicrobial agent were presented with elevated MIC values of 4 or 8 mg/L, just below the EUCAST breakpoint of ≥16 mg/L. This particular result may indicate a shift to higher MICs and possible resistance to this antimicrobial combination if it is used extensively in a potential endemic carbapenemase setting and should be further monitored.

It is also of interest that according to the resistome analysis, differences were detected between the Group A/ST11 and the Group B/ST39 isolates regarding other beta-lactamase genes. Isolates of Group B harboured a considerably higher number of beta-lactamase genes, than the Group A ones. Nevertheless, these differences did not correspond to phenotypic differences among the isolates; all strains from both groups were fully resistant to all beta-lactam antibiotics, with the sole sporadic exception being the few meropenem-vaborbactam marginally susceptible isolates among the Group A/ST11. This particular result is presented for the first time in Greece.

Regarding the isolate #17, of interest is the inconsistent lateral flow assay result (OXA-48/KPC/NDM) with the sequencing results (only the *bla*_KPC-3_ gene was identified). Although this assay is considered a robust, sensitive, and specific one [18], there is at least one study [19] reporting CRKP isolates as falsely positive for the NDM and/or OXA-48 enzymes, as was also the case in this study. The authors were speculating that this occurred probably due to the high concentration of the suspension, as pointed out also in the assay insert. We repeated the assay with various inoculum concentrations and in our hands the assay provided the same result (three carbapenemases). In that respect we believe that this is a rare but possible false positive result of the technique and should be considered in future applications. If more similar false positive results do occur, especially among potential CRKP-DC isolates, further investigation would be warranted.

It should be noted that although all three STs detected in this study do not belong in the group of MLST types associated with the hypervirulent group of isolates (mostly ST23, ST65 and ST86, as well as ST101 and ST147) it has been shown that hypervirulent isolates often acquire carbapenemase genes [8]; thus, in a nosocomial environment abundant in carbapenemases (like in many Greek hospitals) the introduction of a hypervirulent susceptible clone may potentially result in a CPKP hypervirulent outbreak.

A limitation of our study was that it was an investigation of an outbreak of CPKP-DC isolates, without paired detailed clinical data (only a limited set of data related to the bacterial isolates were studied). Nevertheless, the important clinical perspective lies in the identification of the underlying resistance mechanisms that are circulating in an ICU, not just on the antibiotic group level (e.g., carbapenem resistance) but on the resistome scale (older and novel beta-lactams including inhibitor combinations, aminoglycoside, tetracycline, quinolone groups), thus globally mapping resistance with a view to aiding clinical decisions.

## 5. Conclusions

In conclusion, although previously CRKP-DC isolates have been detected in Greece, the present study reported an outbreak mainly due to ST11 CRKP-DC isolates harbouring the combination *bla*_NDM-1_/*bla*_OXA-48_ which is a novel situation in our country and should be further monitored.

## Figures and Tables

**Table 1:** Sample Information and immunochromatography results of the 23 *K. pneumoniae* isolates.

No.	Specimen Type	Date	Carbapenem-Resistant Mechanism (by Immunochromatography)
1	Blood	31 October 2022	OXA48—NDM
2	Bronchial secretions	25 October 2022	OXA48—NDM
3	Blood	20 October 2022	OXA48—NDM
4	Urine	2 November 2022	OXA48—NDM
5	Blood	5 November 2022	OXA48—NDM
6	Bronchial secretions	20 October 2022	OXA48—NDM
7	Bronchial secretions	20 October 2022	OXA48—NDM
8	Catheter tip	9 October 2022	OXA48—NDM
9	Sputum	9 November 2022	OXA48—NDM
10	Urine	14 November 2022	OXA48—NDM
11	Urine	17 November 2022	OXA48—NDM
12	Bronchial secretions	24 November 2022	OXA48—NDM
13	Purulent wound exudates	12 January 2023	OXA48—NDM
14	Purulent wound exudates	18 January 2023	OXA48—NDM
15	Bronchial secretions	25 January 2023	OXA48—NDM
16	Purulent wound exudates	25 January 2023	KPC—NDM
17	Bronchial secretions	30 January 2023	OXA48—KPC—NDM
18	Nasal swabs	1 February 2023	OXA48—NDM
19	Bronchial secretions	6 February 2023	KPC—NDM
20	Blood	8 February 2023	KPC—NDM
21	Blood	8 February 2023	OXA48—NDM
22	Bronchial secretions	15 March 2023	OXA48—KPC
23	Purulent wound exudates	5 April 2023	KPC—NDM

**Table 2:** Antibiotic susceptibility testing results.

Νο.	AMC	AN	ATM	Cl	CAZ	CFM	CIP	CRO	CS	CTX	CXM	ETP	FEP	FOS	GM	IPM	LEV	MEM	MNO	MXF	SXT	TEM	TGC	TM	TZP	CT	CZA	IMR	MEV
1	≥32	16	≥64	≤2	≥64	≥4	≥4	≥64	≥16	≥64	≥64	≥8	≥32	≥256	≥16	≥16	≥8	≥16	4	≥8	≥320	≥32	≤0.5	≥16	≥128	≥32	≥16	>32	8
2	≥32	16	≥64	4	≥64	≥4	≥4	≥64	≥16	≥64	≥64	≥8	≥32	32	≥16	≥16	≥8	≥16	8	≥8	≥320	≥32	1	≥16	≥128	≥32	≥16	>32	16
3	≥32	16	≥64	≥64	≥64	≥4	≥4	≥64	≥16	≥64	≥64	≥8	≥32	≥256	≥16	≥16	≥8	≥16	≥16	≥8	≥320	≥32	≥8	≥16	≥128	≥32	≥16	>32	>256
4	≥32	16	≥64	4	≥64	≥4	≥4	≥64	≥16	≥64	≥64	≥8	≥32	≥256	≥16	≥16	≥8	≥16	≥16	≥8	≥320	≥32	1	≥16	≥128	≥32	≥16	>32	4
5	≥32	16	≥64	≥64	≥64	≥4	≥4	≥64	≤0.5	≥64	≥64	≥8	≥32	≥256	≥16	≥16	≥8	≥16	≥16	≥8	≥320	≥32	4	≥16	≥128	≥32	≥16	>32	4
6	≥32	32	≥64	≥64	≥64	≥4	≥4	≥64	≥16	≥64	≥64	≥8	≥32	≥256	≥16	≥16	≥8	≥16	≥16	≥8	≥320	≥32	4	≥16	≥128	≥32	≥16	>32	>256
7	≥32	32	≥64	4	≥64	≥4	≥4	≥64	≥16	≥64	≥64	≥8	≥32	≥256	≥16	≥16	≥8	≥16	≥16	≥8	≥320	≥32	2	≥16	≥128	≥32	≥16	>32	>256
8	≥32	32	≥64	≥64	≥64	≥4	≥4	≥64	≥16	≥64	≥64	≥8	≥32	≥256	≥16	≥16	≥8	≥16	≥16	≥8	≥320	≥32	4	≥16	≥128	≥32	≥16	>32	>256
9	≥32	32	≥64	≤2	≥64	≥4	≥4	≥64	≥16	≥64	≥64	≥8	≥32	≥256	≥16	≥16	≥8	≥16	≥16	≥8	≥320	≥32	1	≥16	≥128	≥32	≥16	>32	>256
10	≥32	16	≥64	4	≥64	≥4	≥4	≥64	≤0.5	≥64	≥64	≥8	≥32	≥256	≥16	≥16	≥8	≥16	8	≥8	≥320	≥32	1	≥16	≥128	≥32	≥16	>32	4
11	≥32	16	≥64	16	≥64	≥4	≥4	≥64	≥16	≥64	≥64	≥8	≥32	≥256	≥16	≥16	≥8	≥16	≥16	≥8	≥320	≥32	1	≥16	≥128	≥32	≥16	>32	>256
12	≥32	32	≥64	4	≥64	≥4	≥4	≥64	≥16	≥64	≥64	≥8	≥32	≥256	≥16	≥16	≥8	≥16	≥16	≥8	≥320	≥32	1	≥16	≥128	≥32	≥16	>32	>256
13	≥32	16	≥64	16	≥64	≥4	≥4	≥64	≥16	≥64	≥64	≥8	≥32	≥256	≥16	≥16	≥8	≥16	≥16	≥8	≥320	≥32	1	≥16	≥128	≥32	≥16	>32	8
14	≥32	32	≥64	16	≥64	≥4	≥4	≥64	≥16	≥64	≥64	≥8	≥32	≥256	≥16	≥16	≥8	≥16	8	≥8	≥320	≥32	1	≥16	≥128	≥32	≥16	>32	8
15	≥32	32	≥64	8	≥64	≥4	≥4	≥64	≥16	≥64	≥64	≥8	≥32	≥256	≥16	≥16	≥8	≥16	≥16	≥8	≥320	≥32	1	≥16	≥128	≥32	≥16	>32	>256
16	≥32	32	≥64	≥64	≥64	≥4	≥4	≥64	≤0.5	≥64	≥64	≥8	≥32	≥256	≥16	≥16	≥8	≥16	≥16	≥8	≥320	≥32	2	≥16	≥128	≥32	≥16	32	32
17	≥32	32	≥64	≥64	≥64	≥4	≥4	≥64	≥16	≥64	≥64	≥8	≥32	128	≥16	≥16	≥8	≥16	≥16	≥8	≥320	≥32	2	≥16	≥128	≥32	≥16	0.5	1
18	≥32	32	≥64	≥64	≥64	≥4	≥4	≥64	≥16	≥64	≥64	≥8	≥32	≥256	≥16	≥16	≥8	≥16	≥16	≥8	≥320	≥32	2	≥16	≥128	≥32	≥16	>32	>256
19	≥32	32	≥64	≥64	≥64	≥4	≥4	≥64	≤0.5	≥64	≥64	≥8	≥32	≥256	≥16	≥16	≥8	≥16	≥16	≥8	≥320	≥32	4	≥16	≥128	≥32	≥16	>32	32
20	≥32	32	≥64	≥64	≥64	≥4	≥4	≥64	≤0.5	≥64	≥64	≥8	≥32	≥256	≥16	≥16	≥8	≥16	≥16	≥8	≥320	≥32	2	≥16	≥128	≥32	≥16	>32	32
21	≥32	16	≥64	16	≥64	≥4	≥4	≥64	≥16	≥64	≥64	≥8	≥32	≥256	≥16	≥16	≥8	≥16	8	≥8	≥320	≥32	1	≥16	≥128	≥32	≥16	>32	>256
22	≥32	32	≥64	≥64	≥64	≥4	≥4	≥64	≥16	≥64	≥64	≥8	≥32	128	≥16	≥16	≥8	≥16	≥16	≥8	≥320	≥32	2	≥16	≥128	≥32	4	4	32
23	≥32	32	≥64	≥64	≥64	≥4	≥4	≥64	≤0.5	≥64	≥64	≥8	≥32	≥256	≥16	≥16	≥8	≥16	≥16	≥8	≥320	≥32	4	≥16	≥128	≥32	≥16	>32	32

AMC: Amoxicillin/Clavulanate, AN: Amikacin, ATM: Aztreonam, Cl: Chloramphenicol, CAZ: Ceftazidime, CFM: Cefixime, CIP: Ciprofloxacin, CRO: Ceftriaxone, CS: Colistin, CTX: Cefotaxime, CXM: Cefuroxime, ETP: Ertapenem, FEP: Cefepime, FOS: Fosfomycin, GM: Gentamicin, IPM: Imipenem, LEV: Levofloxacin, MEM: Meropenem, MNO: Minocycline, MXF: Moxifloxacin, SXT: Trimethoprim/Sulfamethoxazole, TEM: Temocillin, TGC: Tigecycline, TM: Tobramycin, TZP: Piperacillin/Tazobactam, CT: Ceftolozane/Tazobactam, CZA: Ceftazidime/Avibactam, IMR: Imipenem/Relebactam, MEV: Meropenem/Vaborbactam.

**Table 3:** MLST analysis results.

No.	% Identity	ST	*gap*A	*inf*B	*mdh*	*pgi*	*pho*E	*rpo*B	*ton*B	Main Clonal Group
1	100%	ST11	*gap*A_3	*inf*B_3	*mdh*_1	*pgi*_1	*pho*E_1	*rpo*B_1	*ton*B_4	CG10011
2	100%	ST11	*gap*A_3	*inf*B_3	*mdh*_1	*pgi*_1	*pho*E_1	*rpo*B_1	*ton*B_4	CG10011
3	100%	ST11	*gap*A_3	*inf*B_3	*mdh*_1	*pgi*_1	*pho*E_1	*rpo*B_1	*ton*B_4	CG10011
4	100%	ST11	*gap*A_3	*inf*B_3	*mdh*_1	*pgi*_1	*pho*E_1	*rpo*B_1	*ton*B_4	CG10011
5	100%	ST11	*gap*A_3	*inf*B_3	*mdh*_1	*pgi*_1	*pho*E_1	*rpo*B_1	*ton*B_4	CG10011
6	100%	ST11	*gap*A_3	*inf*B_3	*mdh*_1	*pgi*_1	*pho*E_1	*rpo*B_1	*ton*B_4	CG10011
7	100%	ST11	*gap*A_3	*inf*B_3	*mdh*_1	*pgi*_1	*pho*E_1	*rpo*B_1	*ton*B_4	CG10011
8	100%	ST11	*gap*A_3	*inf*B_3	*mdh*_1	*pgi*_1	*pho*E_1	*rpo*B_1	*ton*B_4	CG10011
9	100%	ST11	*gap*A_3	*inf*B_3	*mdh*_1	*pgi*_1	*pho*E_1	*rpo*B_1	*ton*B_4	CG10011
10	100%	ST11	*gap*A_3	*inf*B_3	*mdh*_1	*pgi*_1	*pho*E_1	*rpo*B_1	*ton*B_4	CG10011
11	100%	ST11	*gap*A_3	*inf*B_3	*mdh*_1	*pgi*_1	*pho*E_1	*rpo*B_1	*ton*B_4	CG10011
12	100%	ST11	*gap*A_3	*inf*B_3	*mdh*_1	*pgi*_1	*pho*E_1	*rpo*B_1	*ton*B_4	CG10011
13	100%	ST11	*gap*A_3	*inf*B_3	*mdh*_1	*pgi*_1	*pho*E_1	*rpo*B_1	*ton*B_4	CG10011
14	100%	ST11	*gap*A_3	*inf*B_3	*mdh*_1	*pgi*_1	*pho*E_1	*rpo*B_1	*ton*B_4	CG10011
**15**	100%	ST11	*gap*A_3	*inf*B_3	*mdh*_1	*pgi*_1	*pho*E_1	*rpo*B_1	*ton*B_4	CG10011
16	100%	ST39	*gap*A_2	*inf*B_1	*mdh*_2	*pgi*_4	*pho*E_9	*rpo*B_1	*ton*B_14	CG10011
17	100%	ST258	*gap*A_3	*inf*B_3	*mdh*_1	*pgi*_1	*pho*E_1	*rpo*B_1	*ton*B_79	CG10011
18	100%	ST11	*gap*A_3	*inf*B_3	*mdh*_1	*pgi*_1	*pho*E_1	*rpo*B_1	*ton*B_4	CG10011
19	100%	ST39	*gap*A_2	*inf*B_1	*mdh*_2	*pgi*_4	*pho*E_9	*rpo*B_1	*ton*B_14	CG10011
20	100%	ST39	*gap*A_2	*inf*B_1	*mdh*_2	*pgi*_4	*pho*E_9	*rpo*B_1	*ton*B_14	CG10011
21	100%	ST11	*gap*A_3	*inf*B_3	*mdh*_1	*pgi*_1	*pho*E_1	*rpo*B_1	*ton*B_4	CG10011
22	100%	ST258	*gap*A_3	*inf*B_3	*mdh*_1	*pgi*_1	*pho*E_1	*rpo*B_1	*ton*B_79	CG10011
23	100%	ST39	gapA_2	*inf*B_1	*mdh*_2	*pgi*_4	phoE_9	*rpo*B_1	*ton*B_14	CG10011

**Table 4:** Resistome analysis of the isolates. Blank squares correspond to absence of the respective gene.

Antibiotic Class	1	2	3	4	5	6	7	8	9	10	11	12	13	14	15	16	17	18	19	20	21	22	23
	ST11	ST11	ST11	ST11	ST11	ST11	ST11	ST11	ST11	ST11	ST11	ST11	ST11	ST11	ST11	ST39	ST258	ST11	ST39	ST39	ST11	ST258	ST39
β-Lactams	*bla*_NDM-1_	*bla*_NDM-1_	*bla*_NDM-1_	*bla*_NDM-1_	*bla*_NDM-1_	*bla*_NDM-1_	*bla*_NDM-1_	*bla*_NDM-1_	*bla*_NDM-1_	*bla*_NDM-1_	*bla*_NDM-1_	*bla*_NDM-1_	*bla*_NDM-1_	*bla*_NDM-1_	*bla*_NDM-1_	*bla*_NDM-1_		*bla*_NDM-1_	*bla*_NDM-1_	*bla*_NDM-1_	*bla*_NDM-1_		*bla*_NDM-1_
																*bla_KPC-2_*			*bla*_KPC-2_	*bla*_KPC-2_			*bla*_KPC-2_
																	*bla*_KPC-3_					*bla*_KPC-3_	
	*bla*_OXA-48_	*bla*_OXA-48_	*bla*_OXA-48_	*bla*_OXA-48_	*bla*_OXA-48_	*bla*_OXA-48_	*bla*_OXA-48_	*bla*_OXA-48_	*bla*_OXA-48_	*bla*_OXA-48_	*bla*_OXA-48_	*bla*_OXA-48_	*bla*_OXA-48_	*bla*_OXA-48_	*bla*_OXA-48_			*bla*_OXA-48_			*bla*_OXA-48_	*bla*_OXA-48_	
	*bla*_OXA-1_	*bla*_OXA-1_	*bla*_OXA-1_		*bla*_OXA-1_	*bla*_OXA-1_	*bla*_OXA-1_	*bla*_OXA-1_	*bla*_OXA-1_		*bla*_OXA-1_	*bla*_OXA-1_	*bla*_OXA-1_	*bla*_OXA-1_	*bla*_OXA-1_			*bla*_OXA-1_			*bla*_OXA-1_		
	*bla*_SHV-182_	*bla*_SHV-182_	*bla*_SHV-182_	*bla*_SHV-182_	*bla*_SHV-182_	*bla*_SHV-182_	*bla*_SHV-182_	*bla*_SHV-182_	*bla*_SHV-182_	*bla*_SHV-182_	*bla*_SHV-182_	*bla*_SHV-182_	*bla*_SHV-182_	*bla*_SHV-182_	*bla*_SHV-182_		*bla*_SHV-182_	*bla*_SHV-182_			*bla*_SHV-182_	*bla*_SHV-182_	
																*bla*_SHV-79_			*bla*_SHV-79_	*bla*_SHV-79_			*bla*_SHV-79_
																*bla*_SHV-89_			*bla*_SHV-89_	*bla*_SHV-89_			*bla*_SHV-89_
																*bla*_SHV-40_			*bla*_SHV-40_	*bla*_SHV-40_			*bla*_SHV-40_
																*bla*_SHV-85_			*bla*_SHV-85_	*bla*_SHV-85_			*bla*_SHV-85_
																*bla*_SHV-56_			*bla*_SHV-56_	*bla*_SHV-56_			*bla*_SHV-56_
	*bla*_CTX-M-15_	*bla*_CTX-M-15_	*bla*_CTX-M-15_	*bla*_CTX-M-15_	*bla*_CTX-M-15_	*bla*_CTX-M-15_	*bla*_CTX-M-15_	*bla*_CTX-M-15_	*bla*_CTX-M-15_	*bla*_CTX-M-15_	*bla*_CTX-M-15_	*bla*_CTX-M-15_	*bla*_CTX-M-15_	*bla*_CTX-M-15_	*bla*_CTX-M-15_	*bla*_CTX-M-15_		*bla*_CTX-M-15_	*bla*_CTX-M-15_	*bla*_CTX-M-15_	*bla*_CTX-M-15_		*bla*_CTX-M-15_
	*bla*_TEM-1B_	*bla*_TEM-1B_		*bla*_TEM-1B_		*bla*_TEM-1B_	*bla*_TEM-1B_	*bla*_TEM-1B_	*bla*_TEM-1B_	*bla*_TEM-1B_		*bla*_TEM-1B_	*bla*_TEM-1B_	*bla*_TEM-1B_	*bla*_TEM-1B_	*bla*_TEM-1B_		*bla*_TEM-1B_	*bla*_TEM-1B_	*bla*_TEM-1B_	*bla*_TEM-1B_		*bla*_TEM-1B_
																*bla*_TEM-207_			*bla*_TEM-207_	*bla*_TEM-207_			*_bla_*_TEM-207_
																*bla*_TEM-230_			*bla*_TEM-230_	*bla*_TEM-230_			*bla*_TEM-230_
																*bla*_TEM-104_			*bla*_TEM-104_	*bla*_TEM-104_			*bla*_TEM-104_
																*bla*_TEM-234_			*bla*_TEM-234_	*bla*_TEM-234_			*bla*_TEM-234_
																*bla*_TEM-217_			*bla*_TEM-217_	*bla*_TEM-217_			*bla*_TEM-217_
																*bla*_TEM-30_			*bla*_TEM-30_	*bla*_TEM-30_			*bla*_TEM-30_
																*bla*_TEM-198_			*bla*_TEM-198_	*bla*_TEM-198_			*bla*_TEM-198_
Aminoglycosides	*aac(3)-IIa*	*aac(3)-IIa*	*aac(3)-IIa*	*aac(3)-IIa*	*aac(3)-IIa*	*aac(3)-IIa*	*aac(3)-IIa*	*aac(3)-IIa*	*aac(3)-IIa*	*aac(3)-IIa*	*aac(3)-IIa*	*aac(3)-IIa*	*aac(3)-IIa*	*aac(3)-IIa*	*aac(3)-IIa*			*aac(3)-IIa*			*aac(3)-IIa*		
																*aac(3)-IId*			*aac(3)-IId*	*aac(3)-IId*			*aac(3)-IId*
			*aac(6′)-Ib*								*aac(6′)-Ib*			*aac(6′)-Ib*		*aac(6′)-Ib*		*aac(6′)-Ib*	*aac(6′)-Ib*	*aac(6′)-Ib*	*aac(6′)-Ib*	*aac(6′)-Ib*	*aac(6′)-Ib*
																*aph(3′)-Ia*	*aph(3′)-Ia*		*aph(3′)-Ia*	*aph(3′)-Ia*		*aph(3′)-Ia*	*aph(3′)-Ia*
	*aph(3′′)-Ib*	*aph(3′′)-Ib*		*aph(3′′)-Ib*		*aph(3′′)-Ib*	*aph(3′′)-Ib*	*aph(3′′)-Ib*	*aph(3′′)-Ib*	*aph(3′′)-Ib*		*aph(3′′)-Ib*	*aph(3′′)-Ib*	*aph(3′′)-Ib*	*aph(3′′)-Ib*	*aph(3′′)-Ib*		*aph(3′′)-Ib*	*aph(3′′)-Ib*	*aph(3′′)-Ib*	*aph(3′′)-Ib*		
	*aph(6)-Id*	*aph(6)-Id*		*aph(6)-Id*		*aph(6)-Id*	*aph(6)-Id*	*aph(6)-Id *	*aph(6)-Id*	*aph(6)-Id*		*aph(6)-Id*	*aph(6)-Id*	*aph(6)-Id*	*aph(6)-Id*	*aph(6)-Id*		*aph(6)-Id*	*aph(6)-Id*	*aph(6)-Id*	*aph(6)-Id*		
																*aadA1*			*aadA1*	*aadA1*			*aadA1*
																*aadA2*			*aadA2*	*aadA2*			*aadA2*
Amphenicols	*catB3*	*catB3*	*catB3*		*catB3*	*catB3*	*catB3*	*catB3*	*catB3*		*catB3*	*catB3*	*catB3*	*catB3*	*catB3*			*catB3*			*catB3*		
																	*catA1*						
Tetracyclines																*tet(M)*			*tet(M)*	*tet(M)*			*tet(M)*
Macrolides																*mph(A)*	*mph(A)*		*mph(A)*	*mph(A)*			*mph(A)*
Quaternary ammonium compound																*qacL*			*qacL*	*qacL*			*qacL*
																*qacE*	*qacE*		*qacE*	*qacE*			*qacE*
Fosfomycin	*fosA*	*fosA*	*fosA*	*fosA*	*fosA*	*fosA*	*fosA*	*fosA*	*fosA*	*fosA*	*fosA*	*fosA*	*fosA*	*fosA*	*fosA*	*fosA*	*fosA*	*fosA*	*fosA*	*fosA*	*fosA*	*fosA*	*fosA*
Quinolones	*OqxA*	*OqxA*	*OqxA*	*OqxA*	*OqxA*	*OqxA*	*OqxA*	*OqxA*	*OqxA*	*OqxA*	*OqxA*	*OqxA*	*OqxA*	*OqxA*	*OqxA*	*OqxA*	*OqxA*	*OqxA*	*OqxA*	*OqxA*	*OqxA*	*OqxA*	*OqxA*
	*OqxB*	*OqxB*	*OqxB*	*OqxB*	*OqxB*	*OqxB*	*OqxB*	*OqxB*	*OqxB*	*OqxB*	*OqxB*	*OqxB*	*OqxB*	*OqxB*	*OqxB*	*OqxB*	*OqxB*	*OqxB*	*OqxB*	*OqxB*	*OqxB*	*OqxB*	*OqxB*
Folate pathway antagonists																*sul1*	*sul1*		*sul1*	*sul1*			*sul1*
	*sul2*	*sul2*		*sul2*		*sul2*	*sul2*	*sul2*	*sul2*	*sul2*		*sul2*	*sul2*	*sul2*	*sul2*	*sul2*		*sul2*		*sul2*	*sul2*		
																sul3			*sul3*	*sul3*			*sul3*
	*dfrA14*	*dfrA14*	*dfrA14*	*dfrA14*	*dfrA14*	*dfrA14*	*dfrA14*	*dfrA14*	*dfrA14*	*dfrA14*	*dfrA14*	*dfrA14*	*dfrA14*	*dfrA14*	*dfrA14*			*dfrA14*		*dfrA14*	*dfrA14*		
																*dfrA12*	*dfrA12*		*dfrA12*	*dfrA12*			*dfrA12*

## Data Availability

The original data presented in the study are openly available in FigShare at https://doi.org/10.6084/m9.figshare.30509924.v1 (accessed on 2 November 2025).

## Abbreviations

The following abbreviations are used in this manuscript:

CRKP	Carbapenem-resistant *Klebsiella pneumoniae*
ST	Sequence type
bla	Beta-lactamase gene
ICU	Intensive Care Unit
MALDI	Matrix-assisted laser desorption/ionization
MIC	Minimum Inhibitory Concentration
MLST	Multi Locus Sequence Typing
cgMLST	Core Genome Multi Locus Sequence Typing
NDM	New Delhi metallo-beta-lactamase
OXA	Oxacillinase beta-lactamase
KPC	*Klebsiella pneumoniae* carbapenemase
VIM	Verona integron-encoded metallo-beta-lactamase
IMP	Active-on-imipenem carbapenemase
